# Atrophie cérébelleuse post accident d’électrisation se manifestant par un état d'agitation psychomotrice: à propos de 2 cas

**DOI:** 10.11604/pamj.2015.20.348.6551

**Published:** 2015-04-10

**Authors:** Marcellin Bugeme, Olivier Mukuku, John Makong Kiji, Bienvenu Mukuku Ruhindiza, Emmanuel Muyumba

**Affiliations:** 1 Centre Neuropsychiatrique Dr Joseph Guislain/Frères de la Charité, Lubumbashi, République Démocratique du Congo; 2 Faculté de Médecine, Université de Lubumbashi, République Démocratique du Congo; 3 Faculté de Médecine, Université de Ngozi, Burundi

**Keywords:** Atrophie cérébelleuse, électrisation, agitation psychomotrice, complications neuropsychiatriques, cerebellar atrophy, electrocution, psychomotor agitation, neuropsychiatric complications

## Abstract

Les accidents d’électrisation (AE) sont relativement fréquents dans la vie quotidienne. L'atrophie cérébelleuse représente une complication rare. Nous rapportons deux cas d'atrophie cérébelleuse qui se sont manifestés révélés par un état d'agitation psychomotrice. L'atteinte du cervelet était bilatérale chez le premier tandis que chez le deuxième, elle était unilatérale droite associée à celle du cerveau droit. La particularité que présentent nos deux observations est non seulement la rareté de l'atrophie cérébelleuse dans les complications neurologiques post AE mais aussi par son mode d'expression clinique.

## Introduction

Les accidents dus au courant électrique sont fréquents aussi bien dans le milieu professionnel que domestique et peuvent concerner toutes les tranches d’âge. Le passage du courant électrique dans l'organisme déclenche un ensemble de manifestations physiopathologiques qui définissent l’électrisation [1]. L'importance d'accidents d’électrisation (AE) réside dans leurs effets sur tous les systèmes de l'organisme et en particulier les systèmes cardiovasculaire, nerveux, musculo-squelettique et le tissu cutané. La localisation, la gravité et le délai de survenue de ces effets sont relativement aléatoires [1, 2]. Au niveau du système nerveux, les effets de l’électricité entraînent des manifestations neurologiques nombreuses qui peuvent être centrales ou périphériques, d'apparition immédiate ou retardée et transitoires ou définitives [2]. Diverses complications neurologiques causées par l’électrisation ont été décrites [2–5] mais aucune publication n'a rapporté un cas clinique d'atrophie cérébelleuse à notre connaissance. Nous rapportons deux cas d'atrophie cérébelleuse qui se sont manifestés révélés par un état d'agitation psychomotrice (EAPM). La particularité que présentent nos deux observations est non seulement la rareté de l'atrophie cérébelleuse dans les complications neurologiques post AE mais aussi par son mode d'expression clinique.

## Patient et observation

### Patient 1

**Figure 1:**
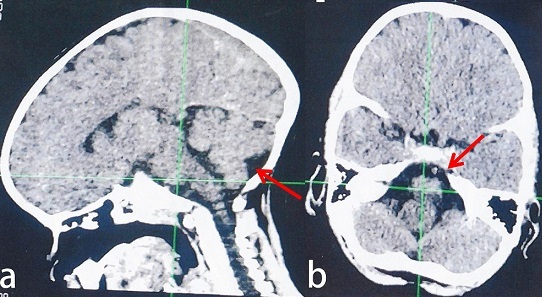
(a et b) images scannographiques montrant des lésions atrophophiques au niveau de l'angle ponto-cérébelleux chez le patient n°1

K.U, âgé de 3 ans, sans antécédents pathologiques, était victime d'un AE par contact direct avec un câble électrique de distribution domestique (220 volts) qui trainait par terre. Ce contact avait projeté l'enfant avec une chute d'une hauteur estimée à 3 mètres, avec notion de perte de connaissance initiale, sans signe d'hémorragie extériorisée. Le patient sera acheminé dans un centre hospitalier où il sera pris en charge en urgence. Quatre mois après l'AE, l'enfant a présenté un trouble de comportement fait de refus de manger, de mélanger la nourriture avec la boue, de refus de communiquer par moment, d'hurlement sans aucune motivation, de nervosité exagérée et d'agressivité avec passage à l'acte. L'examen neuropsychiatrique avait mis en évidence une aphasie motrice, une marche ébrieuse, une agitation psychomotrice et une instabilité. Sur le plan somatique, l'examen avait objectivé des cicatrices de brûlures cutanées siégeant au niveau de la face interne des doigts (index, majeur et annulaire) et celle du pied droit. Aucune lésion cutanée n'avait été objectivée au niveau du crâne. Nous avons conclu à un syndrome cérébelleux associé à un EAPM post AE. Le scanner cérébral avait montré un élargissement du quatrième ventricule et des citernes de base en faveur d'une atrophie de l'angle ponto-cérébelleux (Figure 1). Le tracé d’électroencéphalogramme (EEG) était perturbé par l'abondance en éléments lents diffus. L’électrocardiogramme (ECG) était normal. Le patient avait reçu un traitement fait de rispéridone (1mg deux fois par jour pendant 2 semaines), de chlorpromazine (25mg deux fois par jour pendant 3 jours) et de piracétam (400 mg trois fois par jour pendant deux mois). L’évolution était marquée par une stabilité psychomotrice, une reprise progressive du langage (de l'aphasie vers un langage normal en passant par la dysarthrie) et une marche normale.

### Patient 2

**Figure 2:**
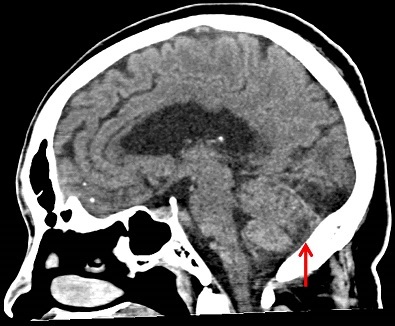
Image scannographique montrant des lésions atropho-cicatricielles au niveau du cervelet chez le patient n°2

Patient T.C, âgé de 54 ans, a été amené au Centre Neuropsychiatrique Dr Joseph-Guislain pour des chutes à la maison et au travail avec perte de connaissance et oublis exagérés. Le début de l'histoire de la maladie remontait à 30 mois de ces manifestations pendant que le patient travaillait dans une station de captation d'eau où il avait touché par imprudence un câble électrique qui transportait 15.000 volts occasionnant chez lui une projection avec perte de connaissance. Il avait été conduit dans un hôpital pour recevoir les premiers soins d'urgence. Notons que le patient n'avait aucun antécédent particulier et, après cet AE, avait évolué pendant plusieurs mois sans poser aucun problème de santé. Vingt-quatre mois après l'AE, le patient a commencé à présenter un trouble de comportement fait d'agitation psychomotrice, de rires immotivés et de propos incohérents par moment. La survenue de crises convulsives afébriles à répétition et d'oublis de façon exagérée avaient motivé sa consultation. Les crises convulsives étaient tonico-cloniques secondairement généralisées, avec révulsion oculaire, émission d’écume et perte d'urine. A la fin des crises, le patient se sentait très fatigué et ne reconnaissait plus rien de ce qui précédait ces crises qui duraient généralement 3 minutes et leur fréquence était de 4 crises par semaine. L'examen neurologique avait mis en évidence une altération des fonctions intellectuelles (altération de la mémoire, d'efficience et d'efficacité et une désorientation temporo-spatiale), une hypermétrie et une adiadococinésie. Le reste de l'examen physique était sans particularité. Nous avons conclu à un syndrome cérébelleux associé à un EAPM post AE. L'EEG et l'ECG étaient normaux. Le scanner cérébral a montré des lésions séquellaires atropho-cicatricielles frontale gauche, occipitale et cérébelleuse droites (Figure 2). Un traitement fait de valproate de sodium (500mg deux fois par jour), de citicholine (2ml trois fois par jour pendant un mois) et de chlorpromazine (50mg deux fois par jour pendant 3 jours) avait instauré. Après 6 mois de suivi médical, l’évolution était marquée, sur le plan psychiatrique, par une diminution progressive de l'agitation et du point de vue neurologique par un arrêt complet des crises convulsives, une amélioration des fonctions intellectuelles et la récupération lente et incomplète de la coordination de mouvements et de l’équilibre.

## Discussion

Les premières descriptions des atteintes neurologiques post AE ont été faites par Silversides en 1964 sur une série de 14 patients exposés à un courant électrique de 220 à 44000 volts [4]. Les complications neurologiques post AE peuvent se manifester soit immédiatement soit après plusieurs heures ou jours, voire plusieurs mois ou années après une exposition électrique [6, 7]. Chez nos patients, le délai d'apparition est de 4 mois pour le premier et d'environ 2 ans pour le second. Les études cliniques les plus approfondies sur les manifestations neurologiques des AE sont faites à partir des accidents post-foudroiement [8] et leurs mécanismes physiopathologiques sont complexes et peu connus. Le courant électrique peut entrainer n'importe quel trouble du système nerveux central, d'apparition immédiate ou retardée, transitoire ou définitive. Le cervelet peut être sélectivement affecté par le courant électrique et son atteinte est rare mais possible. Certains auteurs ont suggéré qu'elle était en rapport avec la vulnérabilité particulière de cette région cérébrale aux variations de température [9]. L'infarctus cérébelleux est la lésion anatomopathologique la plus souvent décrite lors de l'atteinte cérébelleuse après électrisation ou foudroiement [10, 11]. Une dégénérescence rapide du cervelet a été rapportée, avec nécrose des cellules de Purkinje observée moins de 24 heures après le foudroiement [9]. L'atrophie cérébelleuse post AE n'a jamais été décrite auparavant. Nos deux cas cliniques illustrent une complication cérébelleuse, rare, pouvant être rencontrée après un AE. L'atrophie cérébelleuse était bilatérale chez le premier tandis que chez le deuxième, cette atrophie était unilatérale droite associée à celle du cerveau droit. Selon Perdrizet et Zuber, les manifestations neurologiques peuvent être la conséquence du passage du courant électrique lui-même, de la surpression occasionnée (barotraumatismes), d'un traumatisme secondaire à une projection, et d'un arrêt cardiorespiratoire. Toutes les séquelles de l'hypoxie cérébrale peuvent être observées dans ce dernier cas [9]. La substance blanche et les cellules de Purkinje apparaissent particulièrement sensibles à l'hypoxie [12]. Smith souligne que les mécanismes possibles de dommages sévères du tissu nerveux après électrisation peuvent être dus aux dommages thermiques, aux lésions vasculaires ou aux changements électrophysiologiques [13]. L'atrophie cérébelleuse peut être le résultat du passage direct du courant électrique à travers le cervelet et est susceptible de s'aggraver dans les mois suivant l'hyperthermie. Elle paraît être liée directement à l'hyperthermie elle-même, plutôt qu’à l'acidose ou à l'hypoxémie [12]. Dans ce cas, c'est l'action thermique de l’électricité qui est mise en cause. Pour nos deux patients, le mécanisme de survenue de l'atrophie serait multifactoriel. Du point de vue psychiatrique, différents symptômes psychologiques ont été retrouvés à long terme chez les patients victimes d’électrisation [6, 7]. Chez nos deux patients, l'EAPM a constitué le mode de présentation de l'atrophie cérébelleuse. Cette entité psychiatrique a été décrite dans d'autres atteintes neurologiques post AE [5] et dans la série de Silversides, elle a été notée chez 35,7% des cas [4].

## Conclusion

Qu'il soit de basse tension ou de haute tension, le courant électrique peut provoquer des lésions neurologiques graves entre autre une atrophie cérébelleuse. Cette dernière évolue lentement et entraîne un certain degré d'invalidité. D'où une surveillance étroite de victimes d’électrisation s'avère importante.
